# The Effect of *Ginkgo Biloba* (EGb 761) on Epileptic Activity in Rabbits

**DOI:** 10.3390/molecules13102509

**Published:** 2008-10-16

**Authors:** Vesna Ivetic, Mira Popovic, Nada Naumovic, Mirjana Radenkovic, Vesna Vasic

**Affiliations:** 1 Department of Neurophysiology, Medical Faculty, University of Novi Sad, Hajduk Veljkova 3, 21000 Novi Sad, Serbia; E-mails: naumovicnada@hotmail.com (N. N.); v.zaric@yahoo.com (V. V.); 2 Department of Chemistry, Faculty of Sciences, University of Novi Sad, Trg Dositeja Obradovica 3, 21000 Novi Sad, Serbia, E-mail: popovic@ih.ns.ac.yu; 3 Department of Physiology, Medical Faculty, University of Nis, Serbia; E-mail: mirjanakos@metfak.ni.ac.yu (M. R.)

**Keywords:** Kindling epilepsy, *Ginkgo biloba*, EGb 761, Rabbits

## Abstract

Different animal models are used to evaluate the process of epileptogenesis. In this investigation the kindling model of epilepsy was used. The epileptic focus was induced in Chinchilla rabbits by stimulation of the hippocampus with electric stimuli. We presumed that the extracts of *Ginkgo biloba* affect the formation of kindling epilepsy. Bioelectric activity of the brain was registered throughout the development of kindling with and without standardized extracts from dried ginkgo leaves (EGb 761). For each animal the following has been determined: the values of the minimum current strength necessary for the origination of threshold after-discharge (AD) – discharges appearing after the cessation of stimulation; duration of the threshold AD; number of stimulations necessary for the origination of full kindling; time latency for the development of full kindling; number of spontaneous epileptogenic discharges manifested in EEG two days following the formation of full kindling during 60-minute registration. The results show that the process of epileptogenesis was influenced by EGb 761. It has been established that if the animals received EGb 761, significantly weaker minimum current strength was necessary for the development of the epileptogenic focus and the AD were longer, while the number of necessary electrostimulations for the appearance of full kindling was less and the latency was shorter.

## Introduction

Epileptogenesis is a process by which parts of a normal healthy brain are converted to hyperexcitant parts. Different animal models are used to valuate the process of epileptogenesis [1]. To more or less degree, they are all associated with neuronal loss, synaptic reorganization, neurogenesis and gliosis. The kindling model described by Goddard 40 years ago [1,2] has often been used in studying epilepsy. There are different models of kindling like chemical, electrical, etc.

The development of kindling goes on in phases, gradually spreading and reaching brain structures distant from the stimulated area. In the first (development) phase, repetition of stimulations of certain brain regions provokes focal after-discharge activity - AD. AD is the essence of kindling, whereby neuron populations continue to fire in synchronous bursts after the driving stimulation has ceased [6]. This activity is spreading to distant regions and finally may result in motor discharge. In the second (complete) phase each stimulus leads to epileptic discharge, while in the final phase, in animals with formed kindling, the discharge occurs in the lack of electric or chemical stimulation [3,4,5,6,7].

Electrical kindling administration of low intensity daily electrical stimulation to some brain structures induces hyper excitable state and epileptic discharges. With repeated stimulations epileptic discharges grow progressively in duration and complexity. Kindling epilepsy may be provoked in several parts of the brain, but the provocation is the fastest in limbic structures, especially in hippocampus [8,9,10].

Many chemical substances may change the functional state of brain neuron, but it is not known how and to what extent they influence the formation process of kindling state. It is especially interesting whether chemically active principles of plant extracts used as auxiliary medicinal agents may effect epileptogenesis, especially of those plants that have been used worldwide for years due to their influence on the nervous system [11,12].

One of the plants which extracts have shown effect on various neurodegenerative diseases is *Ginkgo biloba* (Gb). We presume that the extracts of this plant may also effect the formation of kindling epilepsy and epileptogenic discharges.

*Ginkgo biloba* L. is a tree species in the Ginkgoaceae (Gymnospermen) family. Many flavonoids have been isolated from ginkgo leaves: monomer flavonoids (quercetin and kaempferol), dimer flavonoids (biflavone, ginkgetin, sciadopitysin and bilobetin), flavonoid glycosides, roanthocyanidines and sterols. Terpene lactones, including the diterpenes ginkgolide A, ginkgolide B, ginkgolide C and the sesquiterpene bilobalide, have also been isolated from ginkgo leaves [13].

Standardized extracts from dried ginkgo leaves (EGb 761) take also important place in modern medicine [14]. A large number of papers indicate neuroprotective effect of Gb and that is why EGb 761 is used both in the prevention and treatment of cognitive disorders and changes during memorizing and remembering process [15,16].

Recently, pharmacological studies (experimental and clinical investigations) have confirmed the therapeutic efficacy of Gb, mainly in cases of microcirculation disturbances [17]. Gb has the effect of anti-aggregation drugs (acting as platelet activating factor antagonists), and free radical scavengers [18,19]. Ginkgo preparations improve the cerebral blood supply and increase tolerance to hypoxia and increase brain levels of ATP and glucose, prevent the origination of nitric oxide, decrease total lactate production by cells and has inhibitory effect on brain lipid peroxidation [20]. It has experimentally been established that Gb phytosome has antioxidant activity; it increases superoxide dismutase, catalase, glutathione peroxidase and glutathione reductase activities in all the brain regions in rats [21].

It has been also shown that it modulates the activity of most neurotransmitters, e.g. catecholamine, serotonin [22] acetylcholine and gamma amino butyric acid – GABA [23], etc.

## Results and Discussion

**Figure 1:**
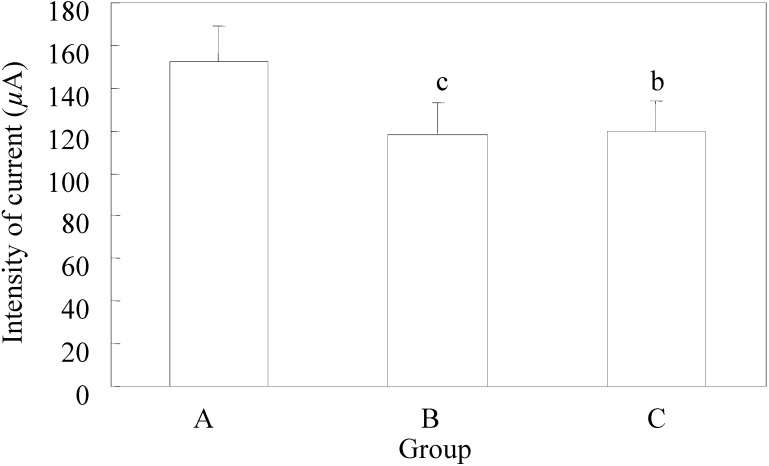
Minimum current strength necessary for threshold after-discharge.

The mean value of the discharged current strength in the group that did not receive EGb 761 – control group was 152 ± 17.3 µA, while in group B it was 118 ± 14.7µA, and in C it was 120 ± 18.8 µA. The mean value of the discharged stimuli strength in each group is presented in Figure 1.

In the control group the lowest value of the discharged strength of stimuli was 120 µA, and the highest was 180 µA, while the lowest values of discharged stimuli strength in groups B and C were equal (100 µA), and the highest values in group B were 140 µA and in group C 160 µA.

The mean values of discharged current strength used in groups B and C did have a significant statistical difference (p=0.48), while the difference related to the first group had a highly significant statistical difference (Figure 1).

A - control group; B - application of EGb 761 in the experiment began five days before the experimental animals were scalped; C - the application of EGb 761 was performed from the day of scalping of each individual experimental animal. n=10; *t* –test ^a^ p<0.05; ^b^ p<0.01; ^c^ p<0.005

**Figure 2:**
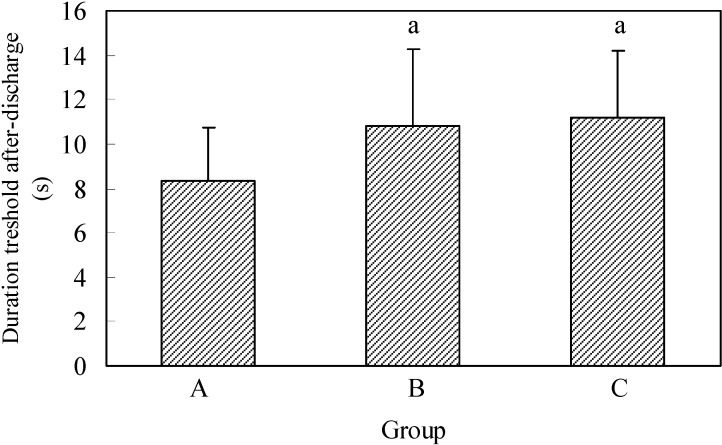
Duration of threshold after-discharge.

In the control group the discharge duration AD lasted the average of 8.4 ± 2.37s, in the second group 10.8 ± 3.48s and in the third group 11.2 ± 2.99s (Figure 2).

The shortest discharge duration time AD in the control group and in group B was 5s, and in animals from group C it was 8s. The longest duration of the first AD in the control group was 12s, in the second 18s and in the third group 17s.

By comparing the duration of threshold AD in the analyzed groups (Figure 2) showed that the average discharge duration AD in the control group had a significantly shorter duration compared with B and when compared with animals in group C.

A - control group; B - application of EGb 761 in the experiment began five days before the experimental animals were scalped; C - the application of EGb 761 was performed from the day of scalping of each individual experimental animal. n=10; *t* –test ^a^ p<0.05; ^b^ p<0.01; ^c^ p<0.005

**Figure 3:**
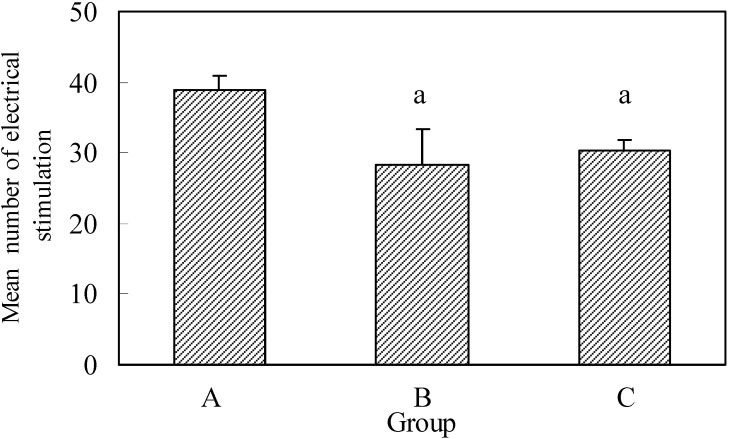
Number of electrical stimulation for development of full kindling epilepsy.

The average number of electrostimulations necessary for the development of kindling was 38.3± 4.5 stimulations in group A, or 1915 electrostimuli. The average number of electrostimulations for the development of full kindling was 1410 electrostimuli in group B, i.e. 28.2 ± 3.04 electrostimulations. In group C there were 1520 electrostimuli during the average of 30.4 ± 1.37 electrostimulations. (Figure 3).

By application of the t test it was established that the number of electrostimulations used necessary for the development of full kindling was statistically higher (p=0.037) in the control group related to group B. A significantly lower number of electrostimulations was applied in group C related to the control group (p=0.048). In group C a lower number of electrostimulations were used than in group B, but difference was statistically not significant (p=0.183) – (Figure 3).

A - control group; B - application of EGb 761 in the experiment began five days before the experimental animals were scalped; C - the application of EGb 761 was performed from the day of scalping of each individual experimental animal. n=10; *t* –test ^a^ p<0.05; ^b^ p<0.01; ^c^ p<0.005

**Figure 4:**
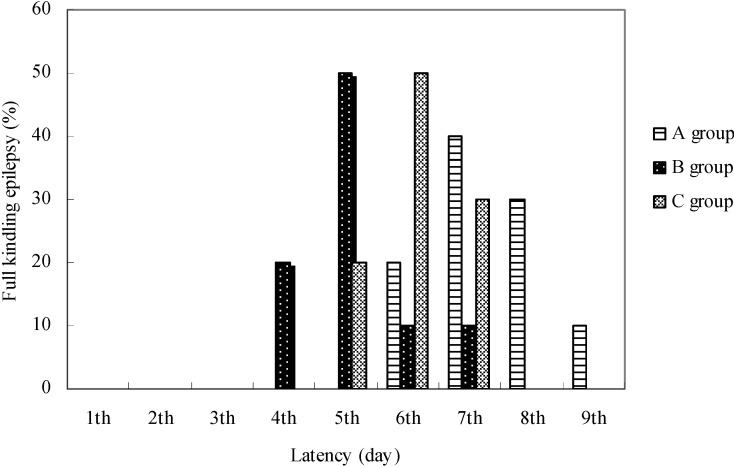
The latent period for development of full kindling epilepsy.

The latency values to develop fully generalized seizures differed in animals of different groups (Figure 4).

A - control group; B - application of EGb 761 in the experiment began five days before the experimental animals were scalped; C - the application of EGb 761 was performed from the day of scalping of each individual experimental animal. n=10.

In the control group the latent period for development of full kindling was six days in 20% of animals, seven days in 40%, eight in 30% and nine days in 10% of animals. According to Racine's seizure classification, in group B two consecutive grade 5 seizures were established in 30% of animals at the end of the 4^th^ electrostimulation day, in 50% on the 5^th^ days, in 10% of the 6^th^ day and in 10% of animals on the 7^th^ day of hippocampus stimulation.

In group C the latent period for the development of full kindling lasted five days in 20% of animals, six days in 50% and seven days in 30% of animals. Full kindling in group B was made in 30% of animals on the 17^th^ day of EGb 761 application, in 50% on the 18^th^ day and in 10% on the 19^th^ and 10% on the 20^th^ day.

In group C full kindling was made in half of the animals on the 14^th^ day of EGb 761 application, and in 30% on the 15^th^ day and in 20% on the 13^th^ day. The number of spontaneous epileptic discharges was determined in the EEG on the second days of the full kindling formation and it was fewer in animals from the control group.

**Figure 5:**
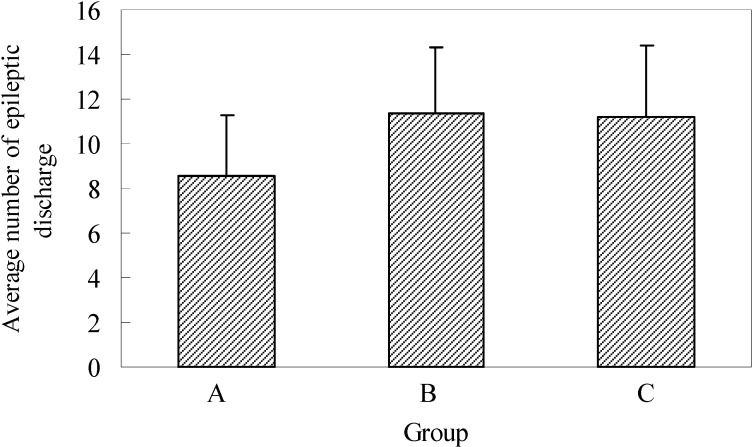
Average number epileptic discharge during 60 min. EEG registration in full kindling.

The average number of epileptogenic discharges visible in EEG, with or without changes in the behavior of experimental animals, established during two-hour registration two days after full kindling had been formed, was 8.6 ± 2.7 in group A, 11.4 ± 2.9 in group B and 11.2 ± 3.2 in group C. The established differences in the number of epileptogenic discharges in control and experimental groups were not statistically significant (Figure 5).

A - control group; B - application of EGb 761 in the experiment began five days before the experimental animals were scalped; C - the application of EGb 761 was performed from the day of scalping of each individual experimental animal. n=10.

The obtained results clearly show that the process of epileptogenesis was influenced by the application of EGb 761 extract. It has been established that if the animals received EGb 761, significantly weaker electric stimuli were needed for the origination of the epileptogenic focus and the AD discharges were longer, while the number of necessary electrostimulations for the origination of full kindling was less and the latency was shorter.

It is especially interesting that the duration of the time interval in which the extract was applied did not significantly affect the course of epileptogenesis, concerning the fact that no statistically significant differences have been established in the values of the observed parameters between animals in groups B and C. In both groups, B and C, were applied EGb 761, but it was 5 days earlier in group B than in group C. In the further investigation, our plan is to prolong this period, i.e. to start application of extract in the group B at least 10 day before than in the group C.

It has been clearly established that the application of the extract had pro-epileptogenic effect, probably by conditioning the depolarization shift of the stimulated neurons of the hippocampus. The same type observations in animal models of epilepsy have already been confirmed before [24].

Thus, by analyzing the convulsive effect of picrotoxin and strychnine it has been established that they are strengthened in ginkgo extract is applied beforehand. It has also been established that some constituents of the extract have pro-epileptogenic effect and decrease the effect of antiepileptic (valproat and carbamazepin) [25,26].

Very important reports show about cases that Gb may precipitate epileptic seizures in patients with well-controlled epilepsy and in those without epilepsy in anamnesis [27,28,29]. The occurrence of epileptic discharges after consuming a large amount of ginkgo nuts has also been described [30]. In this last case generalized convulsions are probably conditioned by the effect of ginkgotoxin (4-*O*-methyl-pyridoxine). Ginkgotoxin functions as a competitive antagonist of pyridoxal phosphate, a coenzyme of glutamate decarboxylase. Depletion of pyridoxal phosphate results in decrease of glutamate decarboxylase activity, decreasing thus also the formation of GABA, so that a ginkgotoxin has pro-epileptogenic effect [31].

In the experimental model used in this research, the pro-epileptogenic effects have probably not been related to ginkgotoxin, as a standardized preparation was used. An attention should be paid to the potential effect of ginkgotoxin and only standardized preparations should be used in daily clinical work, since many patients take EGb 761 extracts in order to improve brain perfusion, improve memory and prevent the development of dementia [32,33,34].

In literature one can find recommendations opposite to our experimental observations, and it is stated primarily in the results of clinical studies that EGb 761 should be used in order to prevent the development of epilepsy due to ischemic attacks, and the protective effect is related to mechanisms of antagonizing platelet activating factor and improving vascularization [18,35]. They presume that antiepileptic effects of Gingko extracts may be partly mediated by bilobalide, a sesquiterpenoid lactone. Bilobalides appear to act at sites in the chloride channel of GABA A receptors and are thus negative allosteric modulators [36,37]. It is also stated that the antiepileptic effect might be related to the flavonoids from EGb 761, for which it has been established that they perform modulation of ionic currents mediated by GABA receptors [38].

Answers to the questions of which are the active principles of EGb 761 and by which mechanisms they influence the threshold of epileptogenic discharges are of interest if we view these fully opposite observations. We think that these variations are caused by non-uniform experimental models, non-uniform dosage and difficulties in precise monitoring of epileptogenic effect, especially when the results of clinical studies are analyzed.

Both epileptogenic and anti-epileptogenic effects of Gb extract are mostly explained by the change of the GABA content, but the change of the functionality of other transmitter systems mediated by e.g. NMDA receptors, beta receptors etc. in various parts of the brain must also be taken into account [39,40]. The obtained results indicate the necessity of further research for precise establishment of the receptor systems by which the extract effect is expressed and establishment of active principles of the extract.

As far as the obtained results are concerned, especially those showing that epileptogenic readiness is increased immediately upon the intake of the extract, one should be cautious in EGb 761 application especially in vulnerable elderly population.

## Conclusions

*Ginkgo biloba* results in pro-epileptogenic effect in animal models. The duration of the time period in which *Ginkgo biloba* is taken does not affect the intensity of its pro-epileptogenic effect.

## Experimental Section

### Biological test

In the research Chinchilla rabbits BW (about 3 kg) were used. The animals were maintained at the Laboratory for Neurophysiology of the Medical Faculty of Novi Sad under standard environmental conditions (i.e. 12:12 h light/dark cycle (07:00 light on), 22°C, with food and water available *ad libitum*). The research was conducted in accordance with the internationally accepted principles for laboratory animal use and care (European Council Directive of November 24, 1986, 86/609/EEC). Animals, randomized in three groups (ten rabbits per group), were allowed to acclimatize for at least 1 week before initiating the experiments.

### Research procedure

The rabbits were anesthetized with 10% novocaine and placed in a stereotaxic frame and positioned to skull-flat position in asepsis and antisepsis conditions. The scalp was clipped off hair and prepared with povidone-iodine (Betadine) before incision.

Five days later implantation with electrodes was made. Sub-cortical electrodes were used. The used electrodes were stainless steel isolated except at the tip. Using a high speed rotational drill, temporal craniotomy was performed, based on the midline sagittal suture, and extending from coronal suture to the lambdoid suture.

Sub-cortical stainless steel stimulating/recording electrode (0.2 mm diameter) was placed stereotaxically into the dorsal hippocampus - in region Ca3. Target coordinates measured from bregma (A-P, 3.0 mm, M-L, +5 mm, and depth 7 mm below dura mater) [41]. All electrodes were secured into place with dental acrylic cement. The animals were allowed to recover two days after electrode placement, before registration of bioelectric activity of the brain (EEG) and before initiating electro stimulation for testing kindling threshold. The registration of the summed bioelectric activity (EEG) was performed monopolarly.

Hippocampal kindling stimulation parameters: the epileptic focus was formed by stimulation of the hippocampus with electric stimuli. Usually at the beginning of the research the discharge threshold after stimulation (after-discharge - AD) was determined. The threshold amplitude defined as the minimal amount of current needed to elicit an AD of >5 s was measured. The threshold AD was determined for each rabbit by stimulating with electrical train (50 Hz with 1.5 ms square-wave for 1 s). One application consisted of 50 electrostimuli. The initial 80 µA current amplitude was increased in 20 µA steps at every following stimulation, until a threshold AD was observed.

Following the determination of the minimal current strength needed for the continuation, AD threshold animals were stimulated six times daily >20 min apart from threshold stimulation. During the research in one experimental animal the hippocampus was stimulated on the same side of the brain.

Behavioral seizure score was analyzed after each kindling stimulation. We used a modified Racine seizure classification (5): stage 1, behavior arrest; stage 2 facial twitching or clonus; stage 3, unilateral forelimb twitching or clonus; stage 4, bilateral forelimb and rearing; stage 5 generalized tonic clonic activities with loss of posture. The animals were stimulated until they achieved two consecutive stage 5 seizures in one day. That day has been taken as the latency to develop fully generalized seizures.

The following has been determined for each animal: the values of the minimum current strength necessary for the origination of threshold AD; duration of the threshold AD; number of stimulations necessary for the origination of full kindling; time latency expressed in days for the development of full kindling; number of spontaneous epileptogenic discharges manifested in EEG two days following the formation of full kindling during 60-minute registration (9-10 h a.m.).

The research included 3 groups with ten animals each. The first group was a control group (A). The animals from other groups received Gb extract EGb 761 (Tanakan Pharma Swiss) of standardized components (40 mg/mL) in a daily dosage of 20 mg/kg body weight divided into three individual doses. The extract was administered every day orally by a dropper at 8 a.m., 2 p.m. and 8 p.m. In the second group (B) with the application of EGb 761 the experiment began five days before the experimental animals were scalped. In the third group (C) the application of EGb 761 was performed from the day of scalping of each individual experimental animal. The application of EGb 761 extract on experimental animals from groups B and C was performed until the day of full kindling formation ending with the evening dosage.

### Histological analysis

The third day from the day following the formation of full kindling - at the end of the research all rabbits were sacrificed with intraperitoneal injections of pentobarbital (Nembutal 150 mg/kg) aiming histological determination of the correctness of the electrode position in the hippocampus. In case of any abnormality, the data obtained from that particular animal were not included in the results.

### Statistical analysis

The obtained results were expressed as the mean X ± SD. ANOVA was used to compare different groups of animals. The p values less than 0.05 were considered to represent a significant difference.
